# Transcriptomic profiling of mice brain under Bex3 regulation

**DOI:** 10.3906/biy-2108-96

**Published:** 2021-11-24

**Authors:** Zhimin XU, Noor BAHADAR, Yingxin ZHANG, Shuang TAN, Zhongze WANG, Bingyi REN, Shitao LIU, Huanyan DAI, Yaowu ZHENG, Bing HAN

**Affiliations:** 1 Department of Oral and Maxillofacial Surgery, School and Hospital of Stomatology, Jilin University, Changchun, China; 2 Key Laboratory of Molecular Epigenetics, Institute of Genetics and Cytology, Northeast Normal University, Changchun, China; 3 Department of Transgenic Animals, WISH Biotechnologies, Changchun, China; 4 Department of Oral Emergency, Hospital of Stomatology, Jilin University, Changchun, China

**Keywords:** CRISPR-Cas9, transgenic mouse model, transcriptome analysis, gene ontology analysis, KEGG pathway

## Abstract

BEX family genes are expressed in various tissues and play significant roles in neuronal development. A mouse model of *Bex3* gene knock-out was generated in this study, using the CRISPR-Cas9 system. Transcriptomic analysis of the brain was performed to identify genes and pathways under *Bex3* regulation. Essential biological functions under the control of *Bex3* related to brain development were identified. Ninety-five genes were differentially expressed under *Bex3^−/−^* regulation, with 53 down and 42 up. Among down-regulated genes, *LOC102633156* is a member of zf-C2H2, *Xlr3a* is an X-linked lymphocyte regulated gene, *LOC101056144* is a hippocampal related gene, *2210418O10Rik* and *Fam205a3* are cortex related genes. Among the upregulated genes, *Zfp967* is a zf protein, *Tgtp2* is a T cell-specific regulator, *Trpc2* is a neuron-related gene, and *Evi2* is related to NF1. A total of 34 KEGG disease terms were identified under the *Bex3^−/−^* regulation. The most prominent is non-syndromic X-linked mental retardation, where *Fgd1* is enriched. Similarly, IRF, MBD, SAND, zf-BED, and zf-C2H2 were significantly enriched transcription factors. A further study is required to confirm and explain each aspect that has been identified in this study.

## 1. Introduction

To date, five BEX genes have been recognized in the genome of humans (BEX1-5), the chimp, the mouse (Bex1-4 and Bex6), while four in the rat genome (Bex1-4). According to the phylogenetic grouping, both mice and rats lack the *Bex5* member of the Bex gene family. Except for *Bex6*, which is found on chromosome 16 of the murine genome, all of the other members are located on the X-chromosome (Alvarez et al., 2005). These genes exhibit moderate sequence homology and are predominantly expressed in the brain (Brown and Kay 1999). A promising characteristic of the BEX genes is that they offer high expression in the mouse brain and are responsible for more than 12% of the rat brain’s expressed sequence tags (Brown and Kay 1999; Alvarez et al., 2005). BEX proteins have a role in transcription control and signalling pathways in neurodegenerative diseases, cell cycle, and tumour growth (Naderi et al., 2012; Zhou et al., 2013; Meng et al., 2014; Fernandez et al., 2015).

*BEX3* is a pro-apoptotic agent facilitated by the neurotrophic receptor p75NTR (Mukai et al., 2003). Others reported a reduction of tumour formation in human breast cancer in *BEX3* xenograft mouse models (Tong et al., 2003). Besides, BEX3-induced downregulation of DRG-1 stimulated PC12 cell proliferation indicates its function in tumour suppression (Yu et al., 2006).

*BEX3* is universally expressed in all human tissues (Uhlen et al., 2015). It is suggested that the BEX3 may be involved in various types of cancers (Krizman et al., 1999). The tumour suppressor role of BEX3 protein was discovered in a xenograft MDA-MB-231 cancer cell model in mice. The overexpression of *mBex3* significantly reduced the tumour formation while compared to the control cells (Tong et al., 2003). This pro-apoptotic characteristic of the *mBex3* was projected by its interaction with p75NTR (Mukai et al., 2000). However, since the p75NTR interaction was not seen in the human models, and the rat *Bex3* showed its interaction with TrkA instead, the function of the *Bex3* is controversial (Tong et al., 2003; Alvarez et al., 2005; Calvo et al., 2015). In recent years, with the rapid development of high-throughput sequencing technology RNA sequencing, has been widely used for transcriptomic analysis (Denoeud et al., 2008). Previously, we have applied this technology to study the lung and brain transcriptomes under the control of different genes (Oo et al., 2020; Sah et al., 2020). In this study, a mouse model of *Bex3* KO was generated using the CRISPR Cas-9 system. The brain transcriptome analysis was performed. We found that several brain-related terms were enriched, indicating the potential biological functional role of *Bex3* in the brain.

## 2. Materials and methods

### 2.1. Housing of the animals

The Institutional Animal Care Committee and Animal Experimental Ethics Committee of NENU have approved the study with the endorsement number (NENU/IACUC, AP2018011). All the recommendations for The Use of Laboratory Animals of NIH, USA, are followed strictly. Mice were housed in IVC cages (5–6 each cage) at rotations of the 12/12 light-dark cycle in a pathogen-free atmosphere with free access to food and water.

### 2.2. sgRNAs designing and plasmids construction

The CRISPR Cas9 system is based on the 20bps nucleotide complementarity (Cong et al., 2013), termed as sgRNAs, followed by a three bps nucleotides, NGG, termed as Protospacer Adjacent Motif (PAM) (Jinek et al., 2013). Benchling database tool (https://benchling.com), was used to design sgRNAs. The sgRNAs designed and the respective positions on the genomic loci are shown in Table 1. The sgRNAs were authenticated about off-target effects using the BLAST tool of NCBI (https://blast.ncbi.nlm.nih.gov/Blast.cgi). The plasmid pX330 (px330-U6-Chimeric_BB-CBhSpCas9, collected from Feng Zhang through Addgene, plasmid# 42230) was linearized using *BbsI*. Two pairs of the sgRNAs, *Bex3*_T1F and *Bex3*_T1R and *Bex3*_T2F and *Bex3*_T2R, were annealed accordingly and ligated to the gel-purified pX330 plasmid. The ligation was double confirmed with *BbsI* restriction digestion and Sanger sequence (Fig S1). These plasmids were named *Bex3*_T1 and *Bex3*_T2. The potential off-targets were identified for each sgRNA, designed the primers to amplify the respective fragments by PCR, and sequenced accordingly (Figures S2 and S3).

### 2.3. Microinjection and genotyping

**Figure 1:**
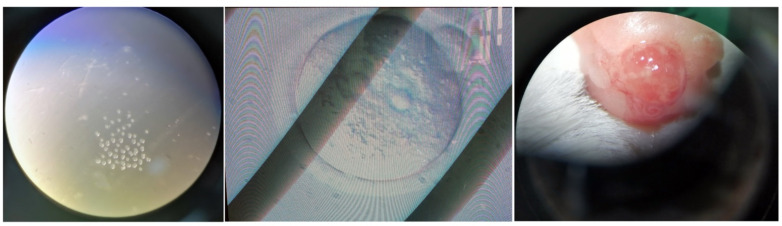
Generation of *Bex3* KO mice. (A) Fertilized eggs extracted. (B) Single-cell oocyte shown with nuclei. (C) Transfer of injected oocytes to the pseudo-mother’s uterus.

All the prerequisites were performed. Maximum eggs (oocytes) were extracted from each mouse, collected in M2 medium plates (Figure 1A). The oocytes were incubated at 37 °C for 2–4 h in the M16 media (Sigma, USA). Similarly, the plasmid vectors were directly injected into the pronuclei of each oocyte following the standard protocol (Figure 1B). After injection, the oocytes were incubated for 4–6 h at 37 °C and were transferred directly to the pseudo-pregnant CD1 females (Figure 1C). Olympus IX71 inverted microscope, and Narishige microinjector was used.

*Bex3* specific primers targeting the upstream and downstream of sgRNAs were designed manually. The PCR was carried out according to the prescribed protocol (Oo et al., 2020). GelDoc was used to observe the gel pictures (Malumbres et al., 1997). The transgenic littermates were separated accordingly. The chimaeras were obtained by crossing the littermates.

### 2.4. Gene expression analysis

Total RNA extraction was performed according to the prescribed instructions using Trizol reagent (Takara, Dalian, China) from the brain of *Bex3^−^*^/^*^−^* and WT. The cDNA was synthesized using a reverse transcription kit following the manufacturers’ instructions (Takara, Dalian). RT-qPCR was conducted with SYBR green mix in triplicates (Takara, Dalian, China), following previous protocols (Oo et al., 2020). GraphPad Prism 8 was used to draw the graphs.

### 2.5. Library preparation for transcriptome analysis

Total RNA from the brain of *Bex3^−/−^* and wild-type mice (each, n=3) was extracted (pooled transcripts) and followed by a further step of DNase I treatment to remove the genomic DNA contamination. The quality of the extracted RNA was tested on Nanodrop, NanoDropTM One spectrophotometer (ThermoFisher Scientific, USA), and Agilent 2100 Bioanalyzer (Santa Clara, USA). One μg of RNA for each transcript was used for the RNA-seq library. RNA-seq was conducted on the BGISEQ-500 platform with paired-end reads. Clean reads were assigned to the mouse genome (GCF_000001635.26_GRCm38.p6) by using the Bowtie2 tool (Langmead and Salzberg 2012).

### 2.6. GO analysis

All the DEGs were assigned to the Gene Ontology (GO) database. To perform GO enrichment analysis, the phyper function in the R program was used. All DEGs were assigned to each term in the Gene Ontology database (http://www.geneontology.org). The number of genes in each term was computed, and the hypergeometric test was employed to find GO terms that are considerably enriched in DEGs equated to the background of all genes in reference species. Bonferroni correction was applied for the p-value (Abdi 2005). Q value (corrected p-value) < 0.05 was determined as significantly enriched GO terms in DEGs.

### 2.7. KEGG analysis

The classification of the KEGG pathway was conducted by assigning all the DEGs to the KEGG pathway database (www.genome.jp/kegg). The pHYPER function in the R program was applied to execute the enrichment analysis to the annotated classification of the KEGG Pathway. Pathway with Q value ≤ 0.05 was defined as significantly enriched in differentially expressed genes (Kanehisa et al., 2008).

### 2.8. Statistical analysis

All the results are shown as means ± SEM (SEM). *P* value <0.05 (unpaired Student’s *t*-test) was considered as statistically significant. All graphs were prepared with GraphPad Prism 8 for Mac (GraphPad Software).

## 3. Results

### 3.1. Strategy of Bex3−/− mouse generation & screening of mutants

**Figure 2:**
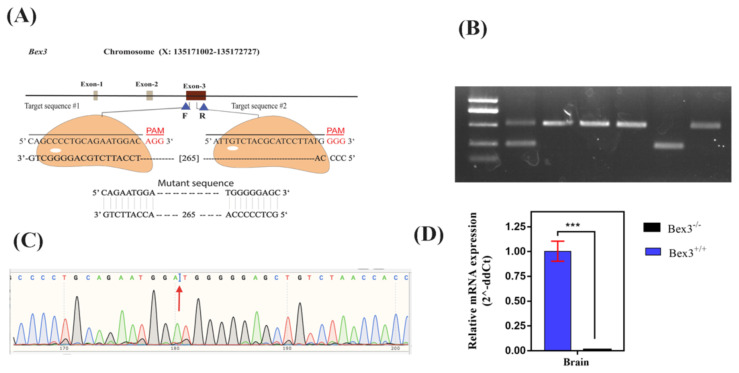
Strategic plan of generating *Bex3* KO mouse model. (A) The coding exon (#3) was targeted for deletion. PAM sequences are shown. (B) A total of 6 pups were obtained; one of them was heterozygous (Pup No. 19), while one was homozygous knocked-out (Pup No. 23). (C) Sanger sequence chromatograph, which shows the repair point after deletion of the fragment. (D) RT-qPCR expression level of mRNA.

The *Bex3* is a small gene of 1726bps, located on the X-chromosome. There are three exons, but only the third exon is coding for the protein. We have targeted third exon. Primers for genotyping were designed flanking the sgRNAs upstream and downstream. A detailed sketch is provided in Figure 2. The px330 plasmid has been used as a vector. The map and design of the plasmid is provided (Figure S5).

Six pups were obtained after 19 days of the oocyte transfer. Fifteen days later to the birth, these pups were weaned, cut their fingers for labelling purposes. The same biopsies were used for genotyping, following the standard protocols. The primers used are shown in Table 2. Two transgenic mice were obtained successfully. The deleted (knocked-out) fragment was confirmed by the Sanger sequence following the standard protocols. These two mice were mated with a wild-type C57BL/6 background to segregate the alleles. F2 progenies were used as experimental organisms.

### 3.2. Brain transcriptome study under *Bex3* regulation

The cDNA libraries were constructed from brain mRNA of seven-weeks-old *Bex3^−/−^* and WT mice in three replicates (n=3). BGISEQ-500 platform was used for RNA-seq. The average yield obtained from *Bex3^−/−^* and WT was 1.18Gb or 23.55M and 23.56M reads for each sample, respectively. Adapter sequences and low-quality reads were filtered out; each sample generated an average of 1.18Gb data, with a Q30 base percentage of 89.33% and 90.59%, while Q20 percentage was 97.43% and 89.77% (Table 3). The clean reads were aligned to the reference genome of the mouse (GCF_000001635.26_GRCm38.p6), and matching competency between clean reads was identified using Bowtie2 (Langmead and Salzberg 2012). Transcript expression levels were computed and presented using RSEM (Li and Dewey 2011). A total of 18,128 genes were detected. The distribution of genes was counted based on three different FPKM cases (Fragment Per Kilo Millions). These three categories were FPKM £1, FPKM1-10, and FPKM³10 (Data not shown). In the first category, about 5362 genes were expressed in *Bex3^−/−^*, and 5311 genes were expressed in WT mice; in the second category, about 6049 genes were expressed in *Bex3^−/−^*, and 6017 genes in WT mice; in the third category, 6717 genes were expressed in *Bex3^−/^*,- and 6800 genes in WT mice were used as control.

**Figure 3:**
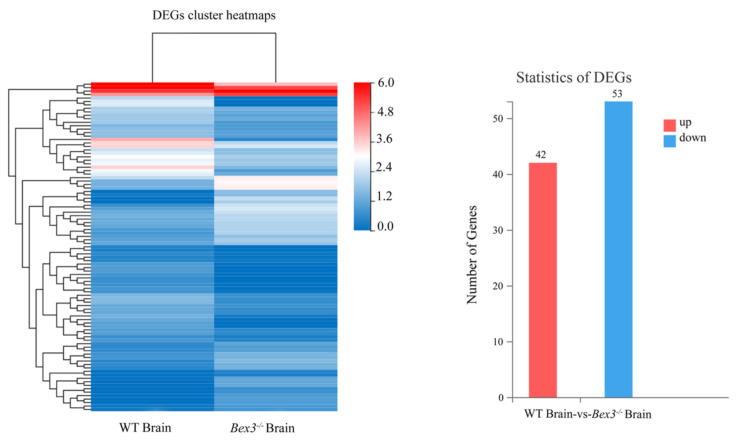
Cluster maps and statistics of DEGs. LEFT: The X-axis denotes the alignment scheme of DEGs for each group, and the Y-axis shows the respective number of differentially expressed genes (DEGs). RIGHT: Red bars represent the number of DEGs upregulated, and blue bars represent the number of DEGs down-regulated.

The genes expressed only in KO mice are termed differentially expressed genes (DEGs). DEGs were identified accordingly (Audic and Claverie 1997). A volcano map of the genes expressed is provided (Figure S4). The criteria set for identification of DEGs was log_2_ fold change > 1, and FDR 0.001. According to the set criteria, a total of 95 unigenes were recognized as expressed differentially between *Bex3^−/−^* and WT, out of which 42 were upregulated, while 53 were down-regulated. Highly significant DEGs are provied (Table S1). A heat map of DEGs is shown in Figure 3. The colour intensity from 0.0 to 6.0 indicates the gene expression level; WT and *Bex3^−^*^/^*^−^* are compared side by side. Moreover, the statistics of the DEGs is provided in the bar graph. The red bar shows the upregulated genes, while the blue bar shows down-regulated genes.

#### 3.2.1. GO enrichment analysis

The Gene Ontology (GO) is a typical gene function classification system that systematically explains the attributes of genes and their products in several organisms. There are three subcategories in the GO annotation: 1) biological process, 2) cellular components, and 3) molecular function (Ashburner et al., 2000). The principal molecular functions identified were GTPase activity, GTP binding, organic acid-binding, etc. (Figure 4A). Under biological process, the enriched terms were cellular response to interferon, defence response to protozoan, adhesion of symbiont to host, cellular response to interferon-gamma, defence response, etc. (Figure 4B). The most enrichment cellular components are symbiont-containing vacuole membrane, haemoglobin complex, Golgi apparatus, etc. (Figure 4C). Other important GO terms, including ‘cation channel complex,’ ‘mesaxon,’ ‘neuronal ribonucleoprotein,’ may also be essential to brain function. We found that most of the GO terms are membrane and extracellular-related, indicating that Bex3 may be necessary for developing neural networks and extracellular signalling.

#### 3.2.2. KEGG pathway analysis

The analysis of the KEGG pathway is used to know the biological function of gene networks. KEGG pathway classification is divided here into five categories: 1) cellular processes, 2) environmental information processing, 3) genetic information processing, 4) metabolism, and 5) organismal systems.

**Figure 5:**
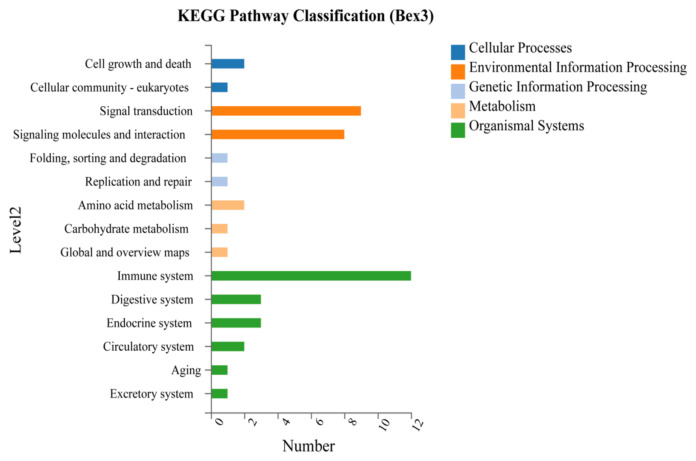
KEGG Pathway classification of DEGs. Y-axis denotes the category of the KEGG pathway, and X-axis represents the number of genes aligned to each class. Blue colour indicates cellular process, orange colour indicates Environmental information processing and sea blue indicates genetic information processing, yellow means metabolism while the green represents organismal systems.

**Figure 6:**
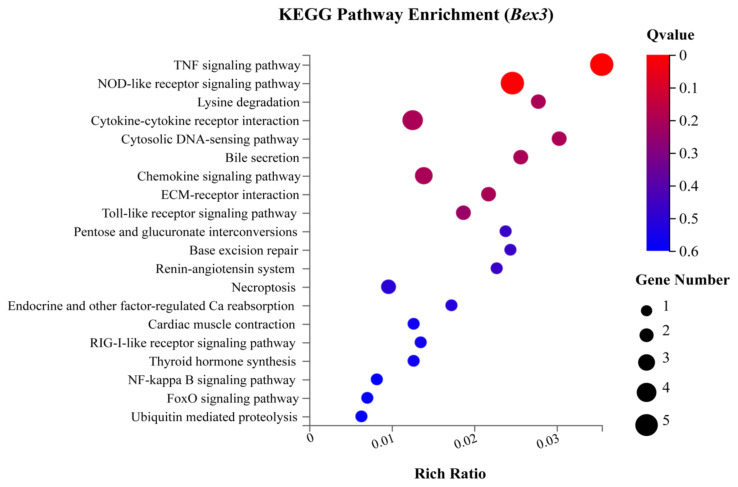
KEGG Pathway enrichment of *Bex3^−/−^*. The X-axis is the enrichment ratio (calculated as Term candidate gene number/Term gene number). The Y-axis is the KEGG pathway term. The size of the circle denotes the number of genes aligned to the KEGG pathway. The colour intensity indicates the enrichment significance.

In the KEGG classification, 31 out of 95 *Bex3^−/−^* regulated DEGs were assigned to multiple pathways from different categories. KEGG Pathway Enrichment Analysis was then conducted to find the significantly enriched pathway in DEGs mapped to the entire genome background. Figure 5 shows five different processes (marked with colours) with selected items. The most significantly enriched pathways with the defined set of criteria were TNF signalling pathway, NOD-like receptor signalling pathway, Lysine degradation, Cytokine-cytokine receptor interaction, etc. (Figure 6). A brief description is provided in Table S2.

#### 3.2.3. KEGG disease-associated pathway analysis

**Figure 7:**
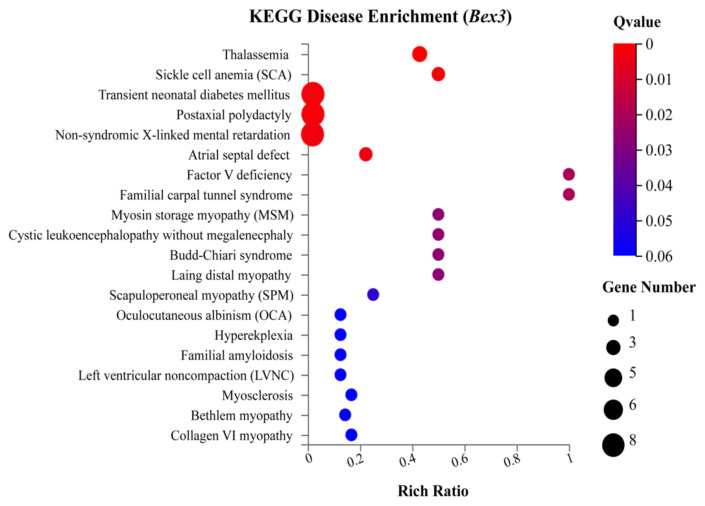
KEGG Disease enrichment. The x-axis represents the enriched terms the y-axis represents the rich ratio. The size of the circle indicates the number of genes. The colour intensity indicates the Q value.

The identified DEGs were assigned to KEGG disease enrichment. Brief description is provided (Table S3). The KEGG disease enriched under *Bex3^−/−^* regulation were thalassemia, sickle cell anaemia, transient neonatal diabetes mellitus, postaxial polydactyl, non-syndromic X-linked mental retardation, and others (Figure 7).

#### 3.2.4. Encoding transcription factor proteins

**Figure 8:**
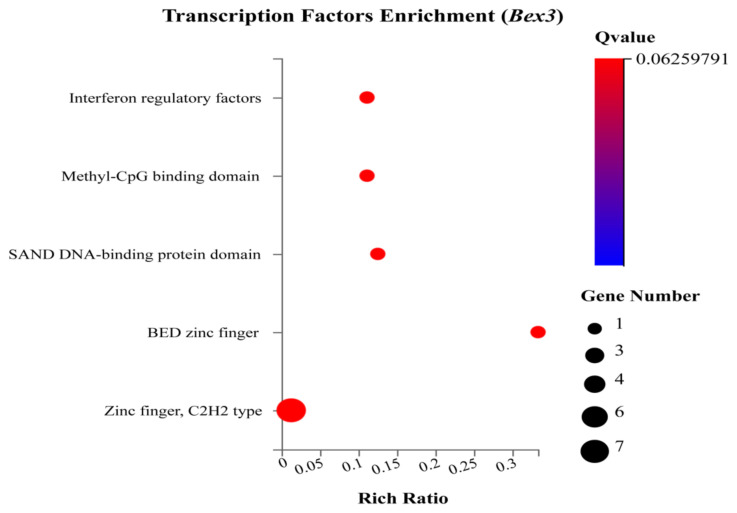
KEGG Transcription Factors enrichment. The x-axis denotes the enriched terms the y-axis represents the rich ratio. The size of the circle indicates the number of genes. The colour intensity indicates the Q value.

The essential regulatory proteins are termed transcription factors that play a role in multiple biological processes. The TFs enriched under *Bex3^−/−^* were interferon regulatory factors, Methyl-DpG binding domain, SAND DNAbinding protein domain, BED zinc finger, zinc finger, C2H2 type transcription factors (Figure 8).

#### 3.2.5. Validation of DEGs via qRT-PCR

**Figure 9:**
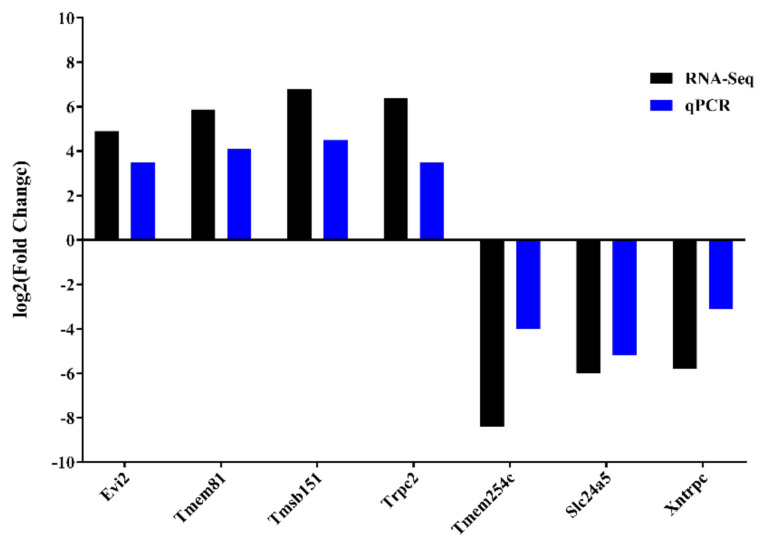
Validation of DEGs using qRT-PCR. The black bars indicate RNA-seq while the blue ones indicate the qRT-PCR results.

To verify the reliability of RNA-seq data, certain DEGs were randomly selected and subjected to qRT-PCR analysis. The expression level of these genes was found consistent. The primers are provided (Table S4) The selected genes were *Tmem254c*, *Slc24a5*, *Xntrpc*, *Evi2*, *Tmem81*, *Tmsb151*, and *Trpc2*. qRT-PCR was performed as explained earlier (Oo et al., 2020). The results are shown in the Figure 9.

## 4. Discussion

RNA-guided genome manipulation based on type II prokaryotic CRISPR/Cas system can be effectively used for gene modification (Jinek et al., 2013; Shen et al., 2013). Using sgRNA, Cas9 can be programmed to catalyze DSB at any targeted site defined by the sgRNA sequence followed by a PAM (Jinek et al., 2013). Plasmid constructions consist of sgRNA, and mRNA encoding Cas9 has been directly injected into embryos to quickly generate transgenic mouse models with various altered alleles (Shen et al., 2013; Wang et al., 2013). Thus, the CRISPR-Cas9 holds massive assurance for editing organisms that are otherwise genetically stubborn.

In this study, a *Bex3^−/−^* mice model was generated using the CRISPR Cas9 system. The targeted gene selected from the brain expressed X-linked gene family, based on its importance in multiple physiological functions. All *Bex* members consist of three exons. Only the last exon codes for proteins (Fernandez et al., 2015). Two loci were targeted by designing sgRNAs to delete the coding exon. A brain transcriptome study was conducted in this study using RNA-seq methodology. A total of 95 unigenes were recognized, out of which 42 were upregulated, while 53 were down-regulated. *Tgtp2*, a glucose transporter, is highly expressed, log_2_ FC 8.1. *Tgtp2* was found to be upregulated in spermatocyte-derived GC-2spd(ts) cells under controlled conditions (Kurihara et al., 2017). *Trpc2,* a TRPC subfamily member, is highly expressed with log_2_ FC value 6.4. *TRPC2* is highly restricted to the dendritic tip of vomeronasal sensory neurons (Zufall et al., 2005). The gene has been established to play a role in mature sperm and the vomeronasal sensory system (Yildirim and Birnbaumer 2007). *Evi2*, a highly expressed gene with a log_2_ FC value 4.90. *EVI2* has been shown as a possible candidate in NF1 disease (Cawthon et al., 1990). Neurofibromatosis type 1 (NF1) is one of the most ordinary inherited human disorders. The potential role of *Evi-2* in murine neoplastic disease and the map representation of the human homolog indicates a possible role for *EVI2* in NF1 (O’Connell et al., 1990). Another highly expressed gene, *Gm45935*, with log_2_ FC value 5.88, is involved in Lysine degradation metabolic pathways. *Zbp1* is moderately expressed with log_2_ FC value 3.9. Researchers have identified that *ZBP1* endorses translocation of the β-actin transcript to actin-rich protrusions in primary fibroblasts and neurons (Huttelmaier et al., 2005). *Ccl21a*, with log_2_ FC value 3.6. The CC chemokine *CCL21* is a strong chemoattractant for lymphocytes and dendritic cells in vitro (Chen et al., 2002). Other researchers demonstrated a specific action of the chemokines, *CCL19,* and *CCL21*, delivering a unique paradigm to study HIV-1 latency in vitro (Saleh et al., 2007). *Gbp4*, with log_2_ FC value 2.8, is a multi-function gene. Gbp^chr3^ deficient mice were exceedingly vulnerable to T. gondii pathogenicity, causing a high parasite load in immune organs (Yamamoto et al., 2012). Similarly, there are several important genes among the downregulated genes among DEGs. *Xlr3a*, with log_2_ FC value −6.75, belongs to a new subfamily in the *Xlr* multigene family. Like *Xlrl*, they are upregulated during B-cell terminal differentiation in normal and neoplastic B-cells and cross-hybridize with a message in testis RNA (Bergsagel et al., 1994). *Evi2b*, is down regulated with log_2_ FC value −4.7. EVI2B is a transmembrane protein universally expressed in hematopoietic cells. It was initially discovered as a common virus incorporation site in murine retrovirally-induced leukaemias, indicating that *Evi2b* might be a proto-oncogene (Kaufmann et al., 1999). *Cyren* is down regulated with log_2_ FC value −3.14. Researchers showed that CYREN (cell cycle regulator of NHEJ) is a cell-cycle-specific inhibitor of cNHEJ (Arnoult et al., 2017). *Xlr3*, an important gene, is down regulated with log_2_ FC value of −6.7. A cluster of X-linked genes has been identified to show transcriptional repression of parental alleles in the developing brain. Imprinting these three *Xlr3b*, *Xlr4b*, and *Xlr4c*, genes is independent of X-chromosome deactivation and has a vibrant and dense form of tissue and time specificity (Raefski and O’Neill 2005).

Interferon regulatory factors are proteins that regulate the transcription of interferons. *Irf7* (Log_2_ FC value 1.2) is a multifunctional transcription factor. Aberrant production of type I IFNs is associated with many diseases such as cancer and autoimmune disorders (Tirone et al., 2021). *Setdb2*, a member of the Methyl-DpG binding domain, is responsible for multiple functions, including oncogenic roles. *Zbed6*, a member of the BED zinc finger, is responsible for several physiological functions. Seven transcription factors were enriched in Zinc finger C2H2 type. We found that under biological process, among the enriched terms hypothetically related to brain development and functions are cerebellar granular layer development and olfactory bulb mitral cell layer development. Other important GO terms, including ‘cation channel complex,’ ‘mesaxon,’ ‘neuronal ribonucleoprotein,’ may also be necessary to brain physiology. GO analysis discovered that several terms are membrane and extracellular-related; *Bex3* may be essential for developing neuronal networks and extracellular signalling (Calvo et al., 2015; Navas-Pérez et al., 2020). TNF signalling pathway, NOD-like receptor signalling pathway, Lysine degradation, Cytokine-cytokine receptor interaction, etc. were the most enriched KEGG pathways. Thalassemia, sickle cell anaemia, transient neonatal diabetes mellitus, postaxial polydactyl, non-syndromic X-linked mental retardation, and others were identified KEGG diseases enriched under the control of *Bex3^−/−^*.

## 5. Conclusion

A mouse model of *Bex3* KO was generated in this study. We did not see any phenotypic differences between the transgenic and normal mice. The transcriptome analysis was performed of the brain. Several DEGs identified in this study are crucial for brain physiology. Moreover, these genes play a crucial role in the proper function of the neurons. Ontology analysis indicated its role in the nervous system as well as in the immune system. A further study is required to confirm these results using respective markers.

## Supplementary Materials

### The sgRNAs confirmation of *Bex3*

Figure S1 Confirmation of sgRNAs for *Bex3* mice model Sanger sequencing chromatographs. (A) sgRNA_1 (B) sgRNA_II

### Off target analysis

1. *Bex2* sgRNA_1_Off-target: The designed sequence of sgRNA_1 was ctcttgtcttctaggagaaa. The sequence was assigned to the BLAST database of NCBI, and the following off-targets loci were selected for PCR to identify any potential off-target cleavage. Only those off-target sites were selected for amplification whose similarity was more than 16/16. The same rules are applied to all sgRNAs. Mus musculus strain C57BL/6J chromosome 8, GRCm39
2. *Bex3* sgRNA_1_Off-target: *Bex3* sgRNA_1 sequence was caggaagaccgccctgtggg and *Bex3* sgRNA_2 sequence was tatgggggagctgtctaacc. Since all the off-targets matching were less than 15/15 bases, the off-target amplification was skipped.

The CRISPR Cas9 system is potentially targeting undesired loci, which is termed as off-target breaks. To identify the off-target effects of sgRNAs for *Bex2*, the similarity scores more than 15/15 were selected for PCR amplification and Sanger sequencing. Since the off-targets similarity scores were less than 15/15 for *Bex3*, the PCR amplification and Sanger sequencing were skipped, rather than the deletion of the fragment was relayed based on mRNA level by RT-qPCR as compared with WT mice (n=3).

Figure S2 Off target loci for each sgRNA and primers sequence for PCR amplification.

Figure S3 Off target analysis The potential off target loci were PCR amplified and sanger sequenced. The chromatogram showed no off-target breaks.

### Volcano map of DEGs of *Bex3^−/−^*

Figure S4 Volcano map of the genes expressed *Bex3^−/−^* brain vs WT brain. Red dots indicate up-regulated genes, green dots indicate down-regulated genes, while grey dots indicate non-DEGs.

Figure S5 The map of the plasmid, used as vector.

### Highly significant DEGs regulated under *Bex3^−/^*

**Table S1:** DEGs regulated under *Bex3*

(A). Up regulated			
Gene ID	Gene Symbol	log2 (BEX3KOBrain/BrainWT)	FDR (BEX3KOBrain/BrainWT)
100303732	*Zfp967*	8.603626345	5.56E-43
100039796	*Tgtp2*	8.194756854	1.50E-41
384081	*Gm13249*	7.714245518	4.69E-04
100862324	*LOC100862324*	6.930737338	6.13E-28
399591	*Tmsb15l*	6.894817763	1.41E-04
105245604	*Gm41035*	6.523561956	9.87E-10
22064	*Trpc2*	6.475733431	1.68E-14
101055676	*LOC101055676*	6.357552005	3.26E-14
107303348	*Gm45935*	5.882643049	4.01E-11
74626	*Tmem81*	5.882643049	7.65E-05
101488212	*Evi2*	4.906890596	1.19E-05
58203	*Zbp1*	3.942514505	4.08E-07
18829	*Ccl21a*	3.636624621	7.10E-06
17472	*Gbp4*	2.852825439	5.62E-47
104245	*Slc6a5*	2.807354922	1.66E-04
631323	*Gm12250*	2.708951218	3.49E-06
76074	*Gbp8*	2.502500341	4.62E-04
100040048	*Ccl27b*	2.387304104	2.58E-12
14469	*Gbp2*	2.356002029	2.68E-44
100038514	*Gm11837*	2.345135486	3.99E-04
21822	*Tgtp1*	2.288481612	4.87E-28
16145	*Igtp*	2.232660757	1.50E-31
60440	*Iigp1*	2.100194288	5.65E-18
102639653	*LOC102639653*	2.026800059	1.22E-12
282619	*Sbsn*	1.931613025	2.12E-05
15953	*Ifi47*	1.743224585	7.77E-05
229898	*Gbp5*	1.560539027	7.66E-16
110257	*Hba-a2*	1.526645005	1.30E-80
55932	*Gbp3*	1.447083226	3.10E-11
11540	*Adora2a*	1.394859617	4.27E-07
54396	*Irgm2*	1.391190757	1.62E-12
60533	*Cd274*	1.361336479	1.08E-06
236573	*Gbp9*	1.309855263	1.18E-06
54123	*Irf7*	1.21790503	5.02E-06
68487	*Tmem140*	1.20029865	8.30E-05
21897	*Tlr1*	1.169925001	2.94E-04
100702	*Gbp6*	1.150025444	9.55E-06
17926	*Myoc*	1.127297808	1.91E-13
105246618	*Gm41885*	1.074343655	5.44E-11
22035	*Tnfsf10*	1.064130337	9.53E-04
628900	*Serpina3i*	1.061776198	1.96E-04
229900	*Gbp7*	1.041820176	9.71E-10
**(B). Down-regulated genes**			
667962	*Zfp966*	−8.991521846	9.64E-58
100042355	*Gm10705*	−8.945443836	5.27E-16
100039192	*Tmem254c*	−8.400879436	4.67E-21
102633156	*LOC102633156*	−6.965784285	3.90E-26
22445	*Xlr3a*	−6.754887502	3.81E-08
101056144	*LOC101056144*	−6.727920455	7.39E-10
100504263	*2210418O10Rik*	−6.491853096	1.67E-17
668605	*Gm9265*	−6.491853096	3.80E-08
100862261	*Fam205a3*	−6.426264755	1.42E-19
100041678	*Gm3500*	−6.189824559	1.30E-05
317750	*Slc24a5*	−6	6.90E-06
105244086	*Gm37416*	−5.906890596	5.19E-07
102443351	*Xntrpc*	−5.882643049	4.60E-13
105245383	*Gm40848*	−5.857980995	5.95E-04
100861978	*LOC100861978*	−5.269741875	9.31E-114
103611159	*Gm38667*	−4.95419631	9.88E-07
216984	*Evi2b*	−4.700439718	1.69E-04
78412	*Cyren*	−3.142957954	1.99E-07
108168534	*Gm46714*	−2.938599455	4.54E-04
21648	*Dynlt1b*	−2.713232745	2.17E-12
14067	*F5*	−2.652076697	3.75E-08
15130	*Hbb-b2*	−2.474538511	3.74E-15
15129	*Hbb-b1*	−2.431962641	7.76E-98
22139	*Ttr*	−2.345952949	0
232156	*Slc4a5*	−2.169925001	3.71E-04
68195	*Rnaset2b*	−2	8.99E-10
100039484	*Gm2260*	−1.874469118	1.67E-05
100039503	*Gm2274*	−1.874469118	1.68E-05
11833	*Aqp8*	−1.711494907	6.12E-06
102638047	*LOC102638047*	−1.610053482	2.57E-05
108168162	*Gm43305*	−1.528161601	6.03E-17
239122	*Setdb2*	−1.520832163	2.00E-05
68553	*Col6a4*	−1.502500341	8.49E-04
240028	*Lnpep*	−1.378511623	9.97E-05
93705	*Pcdhgb8*	−1.316259345	3.74E-06
105244980	*Gm40498*	−1.293991218	6.93E-20
100043920	*Fam205a4*	−1.287980763	1.61E-10
100041874	*Gm3558*	−1.275341301	2.33E-05
214922	*Slc39a2*	−1.26529038	6.78E-08
545124	*Tdg-ps*	−1.258402957	6.66E-09
667118	*Zbed6*	−1.238159737	1.22E-04
100043387	*Gm14305*	−1.222392421	3.30E-09
76380	*Cep112*	−1.198779864	3.44E-04
320506	*Lmbrd2*	−1.160313407	4.18E-16
16589	*Uhmk1*	−1.154014034	4.71E-17
241230	*St8sia6*	−1.106915204	4.73E-04
105244999	*Gm40514*	−1.084888898	1.09E-04
226025	*Trpm3*	−1.052594951	1.24E-14
75209	*Sv2c*	−1.03493379	1.79E-06
140781	*Myh7*	−1.008082346	4.28E-07
100039060	*0610010B08Rik*	−1	4.33E-08
16591	*Kl*	−1	1.89E-10
21892	*Tll1*	−1	1.05E-04

### KEGG pathways regulated under *Bex3^−/−^*

**Table S2:** KEGG pathways regulated under *Bex3^−/−^*

Gene Symbol	KEGG Pathway Term	KEGG Pathway Classification
*Tgtp2*	04668///TNF signaling pathway	Environmental Information Processing
*Ccl27b*	04060///Cytokine-cytokine receptor interaction	Organismal Systems
*Gm10705*	04120///Ubiquitin mediated proteolysis	Genetic Information Processing
*LOC100861978*	04060///Cytokine-cytokine receptor interaction	Organismal Systems
*Gm45935*	00310///Lysine degradation	Metabolism
*Adora2a*	04015///Rap1 signaling pathway	Organismal Systems
*Aqp8*	04976///Bile secretion	Organismal Systems
*F5*	04610///Complement and coagulation cascades	Organismal Systems
*Myh7*	04261///Adrenergic signaling in cardiomyocytes	Organismal Systems
*Gbp2*	04621///NOD-like receptor signaling pathway	Organismal Systems
*Ifi47*	04668///TNF signaling pathway	Environmental Information Processing
*Kl*	04961///Endocrine and other factor-regulated calcium reabsorption	Organismal Systems
*Ccl21a*	04064///NF-kappa B signaling pathway	Organismal Systems
*Tgtp1*	04668///TNF signaling pathway	Environmental Information Processing
*Tlr1*	04620///Toll-like receptor signaling pathway	Organismal Systems
*Tnfsf10*	04217///Necroptosis	Organismal Systems
*Ttr*	04918///Thyroid hormone synthesis	Organismal Systems
*Gbp5*	04621///NOD-like receptor signaling pathway	Organismal Systems
*Gbp7*	04621///NOD-like receptor signaling pathway	Organismal Systems
*Slc4a5*	04976///Bile secretion	Organismal Systems
*Setdb2*	00310///Lysine degradation	Metabolism
*Lnpep*	04614///Renin-angiotensin system	Organismal Systems
*Irf7*	04621///NOD-like receptor signaling pathway	Organismal Systems
*Tdg-ps*	03410///Base excision repair Genetic	Information Processing
*Gbp3*	04621///NOD-like receptor signaling pathway	Organismal Systems
*Zbp1*	04217///Necroptosis	Organismal Systems+++Cellular Processes

Note. Several genes are involved in multiple pathways. Here, only the primary involvement is shown.

### DEGs annotated to significant KEGG diseases regulated under *Bex3^−/−^*

**Table S3:** DEGs annotated to significant KEGG diseases regulated under *Bex3^−/−^*

Gene Symbol	KEGG Disease Term	Log _2_
		( *Bex3 ^−/−^*/WT)
0610010B08Rik	H00513///Transient neonatal diabetes mellitus (TNDM)+ H00480///Non-syndromic X-linked mental retardation+ H01852///Postaxial polydactyly	−1
Gm14305	H00513///Transient neonatal diabetes mellitus (TNDM)+ H00480///Non-syndromic X-linked mental retardation+ H01852///Postaxial polydactyly	−1.222392421
Zfp967	H00513///Transient neonatal diabetes mellitus (TNDM)+ H00480///Non-syndromic X-linked mental retardation+ H01852///Postaxial polydactyly	8.603626345
2210418O10Rik	H00513///Transient neonatal diabetes mellitus (TNDM)+ H00480///Non-syndromic X-linked mental retardation+ H01852///Postaxial polydactyly	−6.491853096
LOC102633156	H00513///Transient neonatal diabetes mellitus (TNDM)+ H00480///Non-syndromic X-linked mental retardation+ H01852///Postaxial polydactyly	−6.965784285
LOC102639653	H00513///Transient neonatal diabetes mellitus (TNDM)+ H00480///Non-syndromic X-linked mental retardation+ H01852///Postaxial polydactyly	2.026800059
Gm40848	H00513///Transient neonatal diabetes mellitus (TNDM)+ H00480///Non-syndromic X-linked mental retardation+ H01852///Postaxial polydactyly	−5.857980995
Zfp966	H00513///Transient neonatal diabetes mellitus (TNDM)+ H00480///Non-syndromic X-linked mental retardation+ H01852///Postaxial polydactyly	−8.991521846

**Table S4:** DEGs validation primers To validate the RNA-seq results, we have performed qRT-PCR. The genes selected and their respective primers are given here.

Gene name	Primer name	Primer sequence
*Tmem254c*	F	TGGCTCGGAAACTTGACTCG
	R	ACCAGACCCGGAAGGTTTTG
*Slc24a5*	F	CTTGCTCCCTTCCTGAACCA
	R	TGCAGAGACCCAAAAGATCCC
*Xntrpc*	F	AGAAGACATCGGGGGAGGAA
	R	GTGACTGTCTGGGTTCAGCA
*Evi2*	F	TGCAGCGGAAAGGAAATGTG
	R	GCCACCTCAGTTCTGCTCTT
*Tmem81*	F	GGGCTGTGCAAACTCCCAGT
	R	CTTAAACCCACAGGCTGCATGA
*Tmsb15l*	F	CTTGGAACCGGCAGACAAGA
	R	CGCTCATCTTGTTTTCATTCGAC
*Trpc2*	F	AGTTCAAGCATTCAGCCCCT
	R	TCACAATCTCAGTCCAGTTGGG

## Figures and Tables

**Figure 4A:**
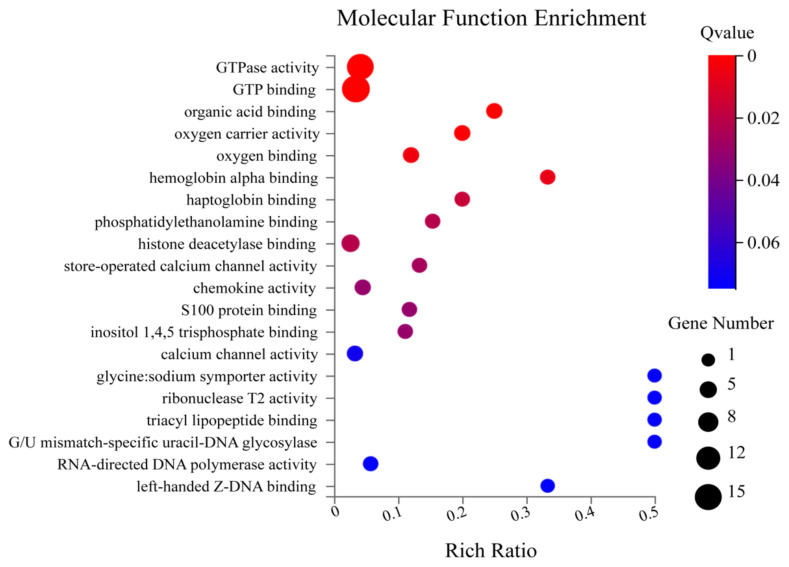
GO Enrichment Molecular Function. The X-axis shows the enriched terms; the Y-axis denotes the rich ratio. The size of the circle represents the gene number. The colour intensity indicates the Q value.

**Figure 4B:**
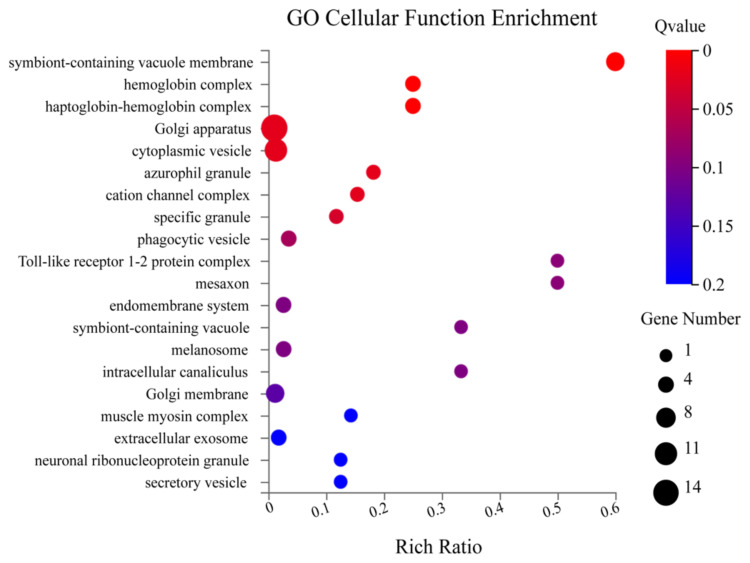
GO Enrichment of Biological Function. The X-axis shows the enriched terms the Y-axis denotes the rich ratio. The size of the circle represents the gene number. The colour intensity indicates the Q value.

**Figure 4C:**
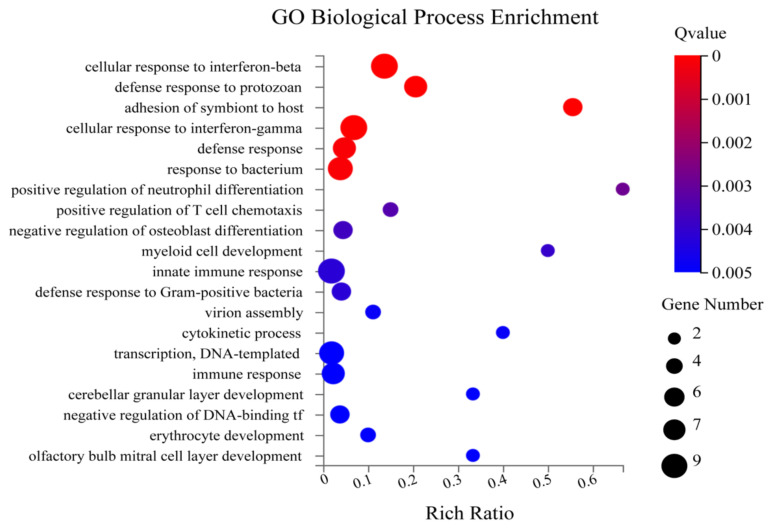
GO Enrichment Cellular Function. The X-axis denotes the enriched terms the Y-axis denotes the rich ratio. The size of the circle represents the gene number. The colour intensity indicates the Q value.

**Table 1:** The sgRNAs positions and the targeted PAM sequences.

sgRNA name	Locus	sgRNA seq	PAM	Remarks
*Bex3_T1*	1092	caggaagaccgccctgtggg	AGG	DSB position 1
*Bex3_T2*	1356	tatgggggagctgtctaacc	ACC	DSB position 2

**Table 2:** Genotype and RT-qPCR primers.

Name	Sequence	Used for
*Bex3* Primer F	ctggtcactgcatcgagcatt	Genotype
*Bex3* Primer R	tacagcgggagtcacagtat	
*Bex3* qF	ccaatgtccaccaggaaaac	RT-qPCR
*Bex3* qR	aggcataaggcagaattcatca	

**Table 3:** Statistics of each gene sequence output.

Name	Over-all Clean Reads (M)	Over-all Mapping (%)	Distinctively Mapped	Q30 (%)	Q20 (%)
WT Brain	23.56	95.46	80.62	90.59	89.77
*Bex3^−/−^* Brain	23.55	95.36	81.29	89.33	97.43
