# Supplementary figures and images for: Transcriptomic profiling of mice brain under Bex3 regulation
Source: Turk J Biol. 2021 Nov 24;46(1):57–68. doi: 10.3906/biy-2108-96 (PMC10393102; doi:10.3906/biy-2108-96)

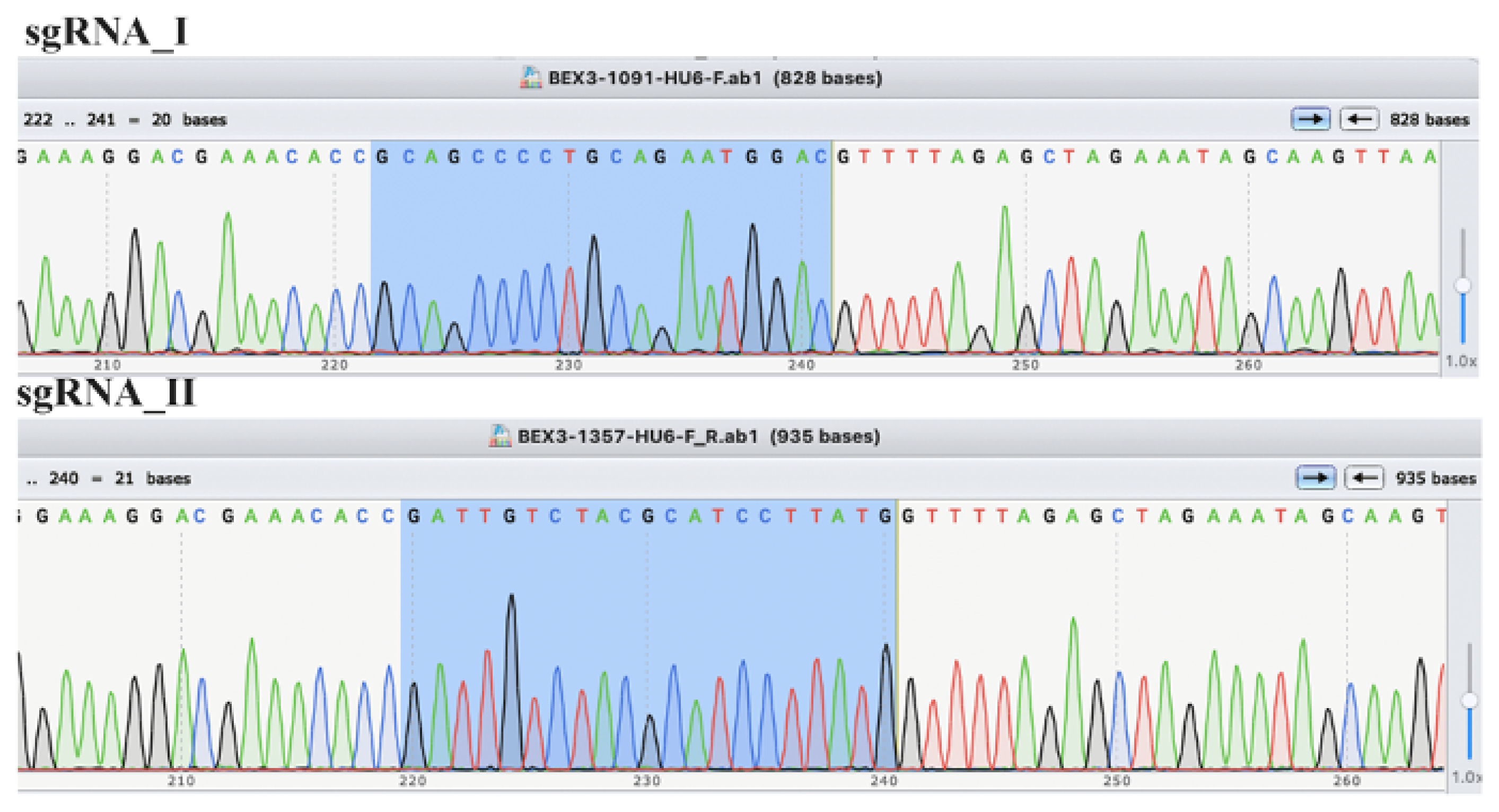

Supplement: Figure S1 — Sanger sequencing chromatographs. (A) sgRNA_1 (B) sgRNA_II [file turkjbiol-46-1-57s1.tif]

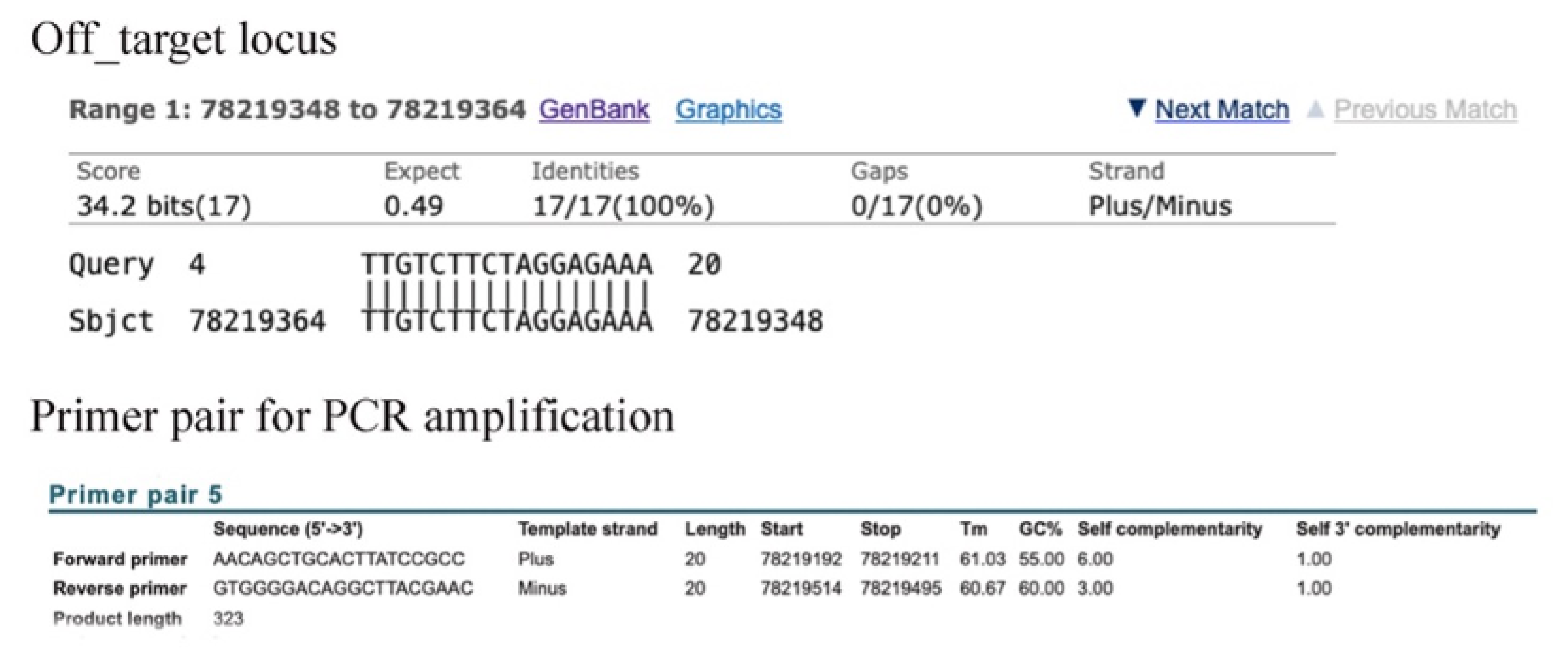

Supplement: Figure S2 — Off target loci for each sgRNA and primers sequence for PCR amplification. [file turkjbiol-46-1-57s2a.tif]

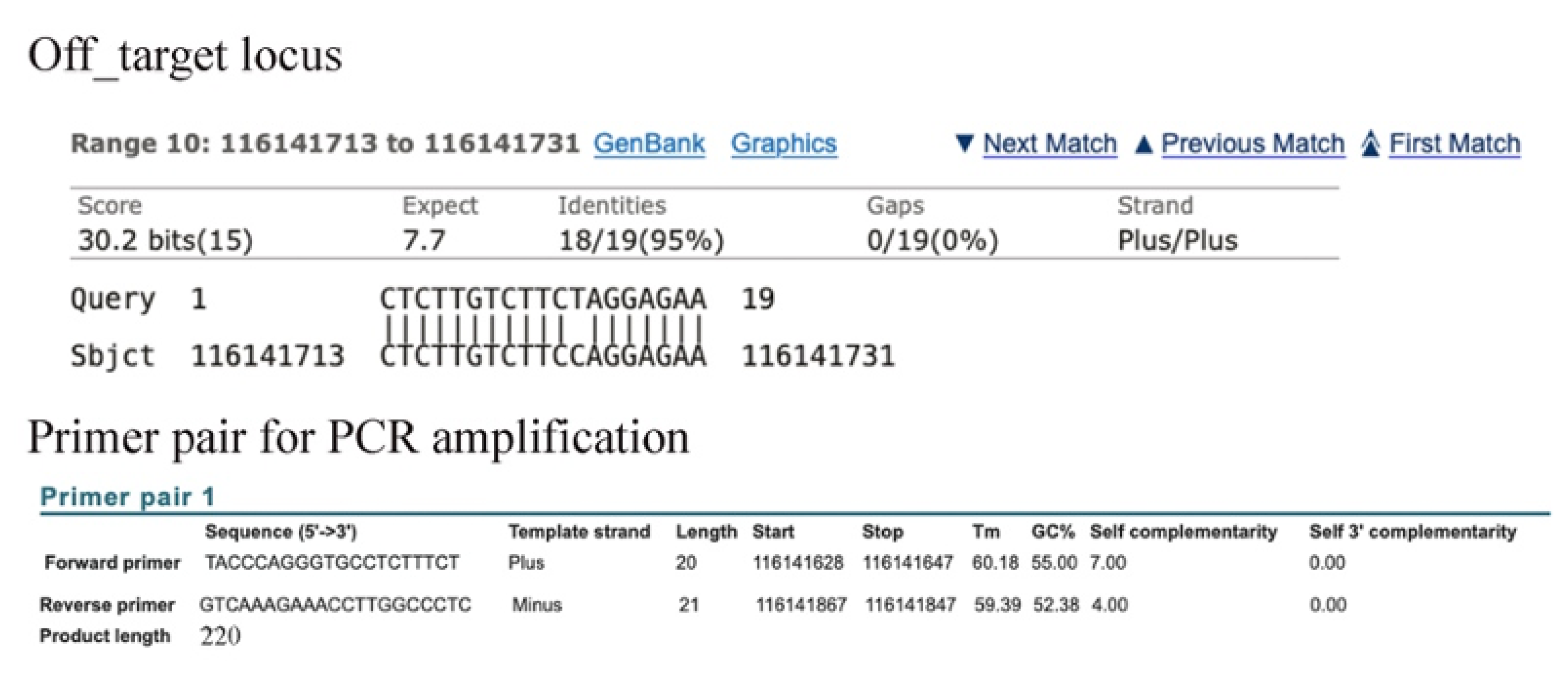

Supplement: Figure S2 — Off target loci for each sgRNA and primers sequence for PCR amplification. [file turkjbiol-46-1-57s2b.tif]

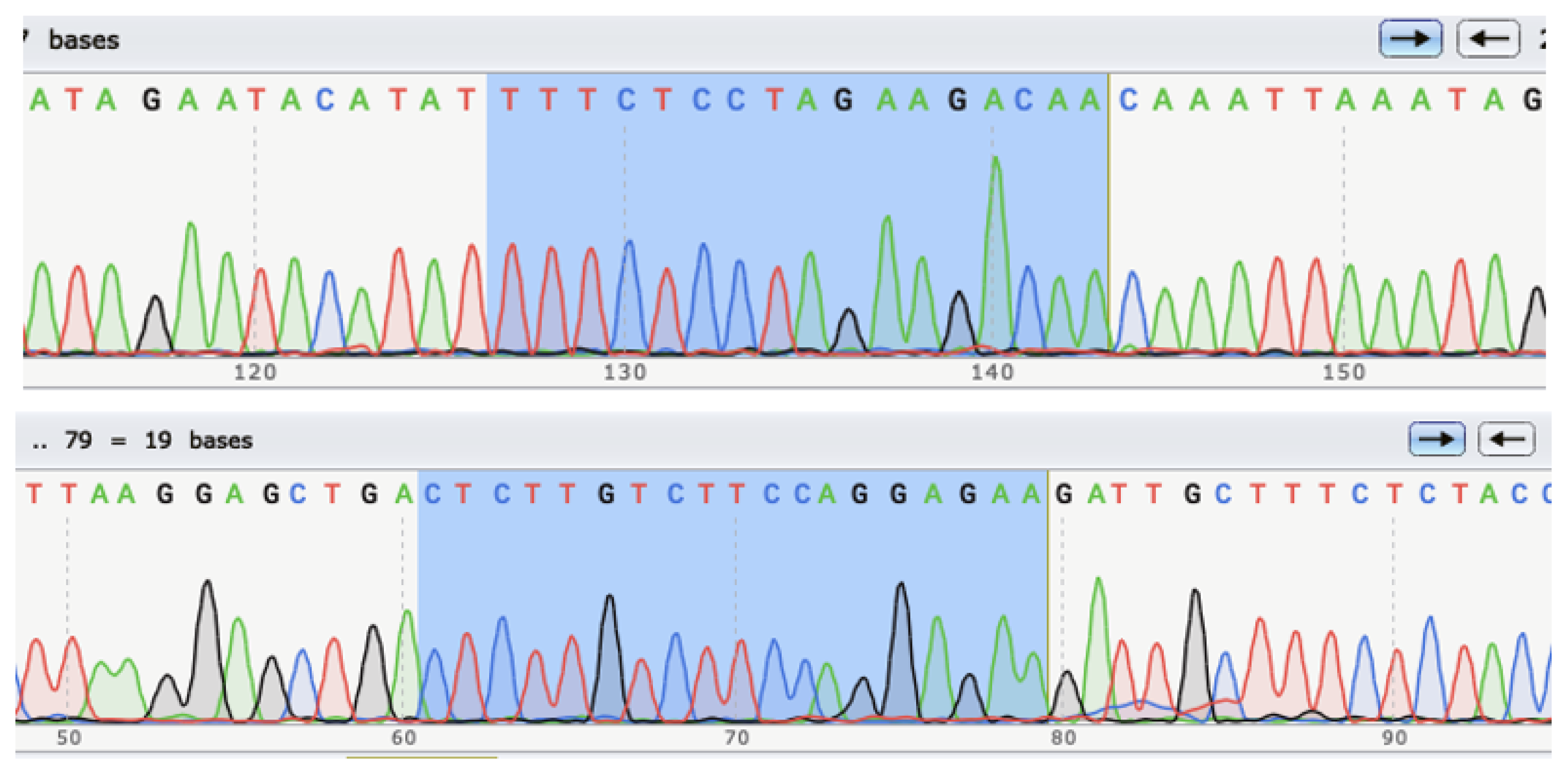

Supplement: Figure S3 — The potential off target loci were PCR amplified and sanger sequenced. The chromatogram showed no off-target breaks. [file turkjbiol-46-1-57s3.tif]

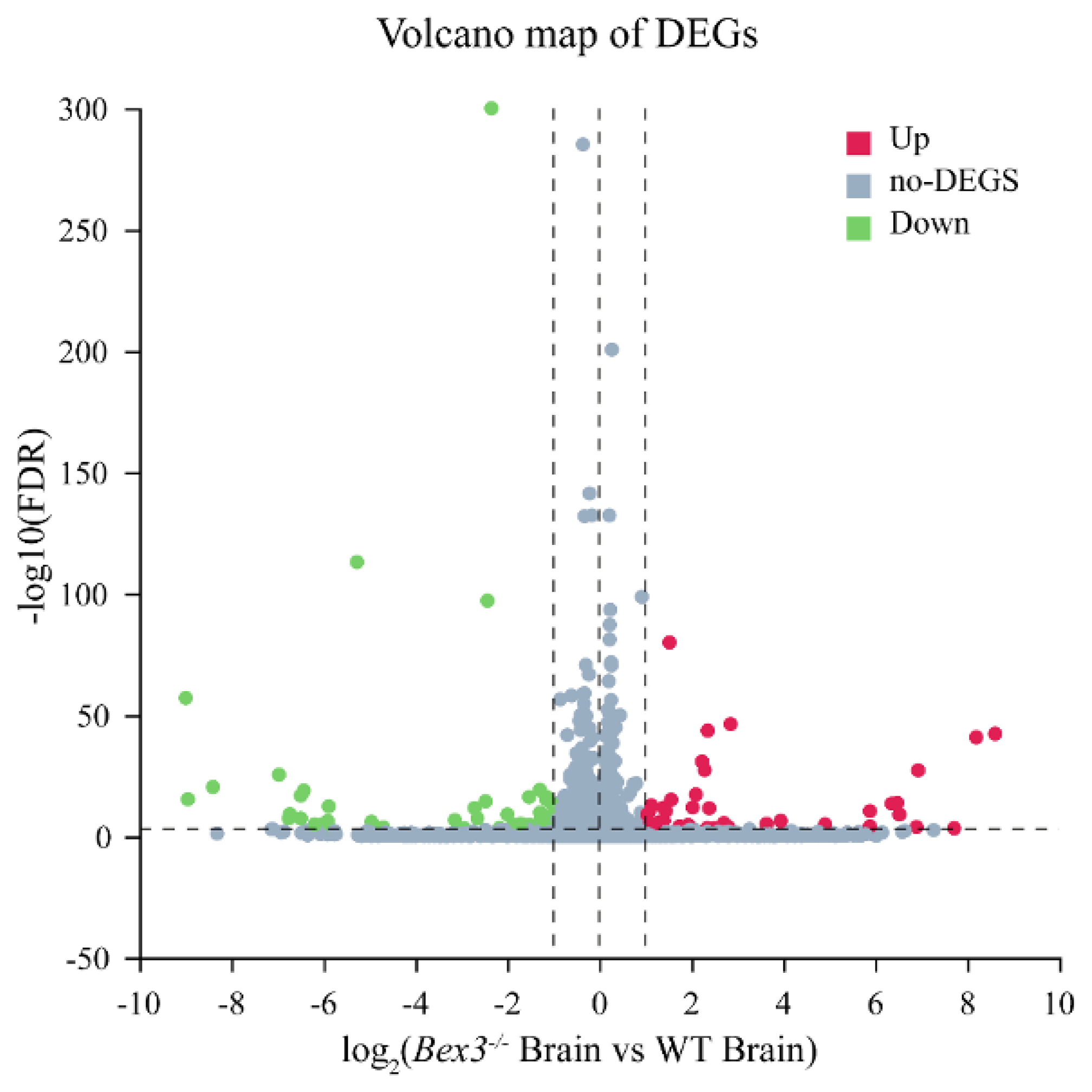

Supplement: Figure S4 — Bex3−/− brain vs WT brain. Red dots indicate up-regulated genes, green dots indicate down-regulated genes, while grey dots indicate non-DEGs. [file turkjbiol-46-1-57s4.tif]

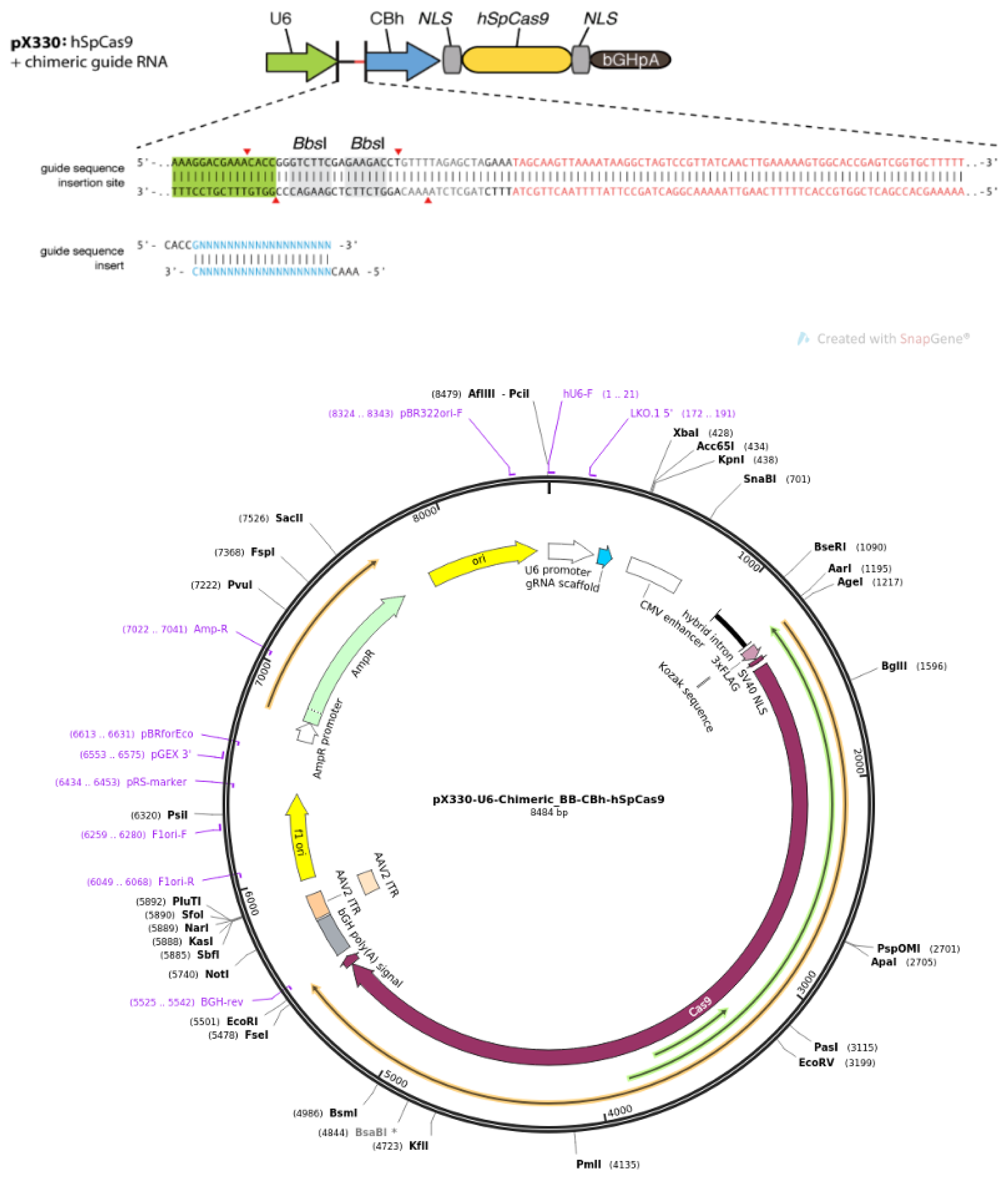

Supplement: Figure S5 — The map of the plasmid, used as vector. [file turkjbiol-46-1-57s5.tif]
